# Assessing the Ecological Effects of Water Transport to a Lake in Arid Regions: A Case Study of Qingtu Lake in Shiyang River Basin, Northwest China

**DOI:** 10.3390/ijerph16010145

**Published:** 2019-01-07

**Authors:** Xunzhou Chunyu, Feng Huang, Ziqiang Xia, Danrong Zhang, Xi Chen, Yongyu Xie

**Affiliations:** 1 State Key Laboratory of Hydrology-Water Resources and Hydraulic Engineering, Hohai University, Nanjing 210098, China; zodiacnix@163.com (X.C.); zqxia@hhu.edu.cn (Z.X.); 20030112@hhu.edu.cn (D.Z.); 20040608@hhu.edu.cn (Y.X.); 2 College of Hydrology and Water Resources, Hohai University, Nanjing 210098, China; xichen@hhu.edu.cn; 3 Institute of Surface-Earth System Science, Tianjin University, Tianjin 300072, China

**Keywords:** ecological effects, spatiotemporal variation, water transport, arid regions, Qingtu Lake

## Abstract

With the continuous growth of economic water consumption in arid regions, many endorheic rivers and terminal lakes have desiccated. As an important ecological engineering measure, water transport in arid regions has vital ecological significance for protecting the regional ecological environment and delaying desertification. In this study, Qingtu Lake, the terminal lake of Shiyang River, was selected to analyze the ecological effects of water transport by means of remote sensing interpretations and current year field investigations. The results demonstrated that, in July 2018, the water surface had formed and recovered to 5.68 km^2^. Additionally, Qingtu Lake formed a spatial gradient distribution in groundwater depth. The depth increased in gradient from the waterside to the desert edge. There was a significant increase in the overall regional vegetation coverage, which mainly occurred in the water areas because of the extensive growth in *Phragmites australis*, which reached 10.54 km^2^ in area in 2018. Furthermore, the regional vegetation formed a gradient distribution, which transitioned from hygrophytes to xerophytes. This study can provide guidelines for the protection and restoration of lakes in arid regions.

## 1. Introduction

Water, as the most crucial ecological factor, is the basis for the formation, development and stability of oasis ecosystems in arid regions [1]. Therefore, the rational development and utilization of water sources in arid regions is extremely important. For endorheic rivers, which are among the key water sources in arid regions, the contradiction between the economy and the environment in the process of water resource development has been prominent for a long time. Specifically, the development of oases in the upper and middle reaches comes at the cost of large-scale desertification of natural oases and shrinkage or drying up of terminal lakes in the lower reaches [2], such as the Tarim River, Heihe River and Shiyang River basins in Northwest China as well as the Aral Sea basin which is located in Central Asia.

In these basins, large-scale development of water and soil resources has been carried out in the 1950s–1960s. During this period, numerous engineering facilities were built to satisfy the irrigation needs of farmlands in the upper and middle reaches [3,4,5]. As a result, surface runoff downstream drastically decreased and directly led to the shrinkage or even drying up of the terminal lakes, which caused a significant degradation of vegetation in the terminal lake areas and led to the aggravated spread of desertification and salinization [6,7]. As an important ecological barrier in arid regions, the terminal lakes suffered almost irreversible damage.

Hence, various measures have been taken to restore the ecosystems of terminal lakes. For the Aral Sea, a 13 km earthen dam that regulated the flow from the Small to Large seas was built in 2005 [8]. The water level of the Small Aral Sea has been kept at about 42 m as of 2006, which restored the local fisheries [5]. While the Kok-Aral Dam can only stabilize the level of the Small Aral, the shrinkage of the Large Aral in the south cannot be prevented. In the Tarim River, ecological water transportation into the lower reaches has been implemented since May 2000 through releases from the Daxihaizi Reservoir planned in a way to keep each release within the river channels [9]. It not only raised the groundwater depth from 9.87 m to 2.66 m after the fourth water delivery [10], but also restored the endangered *Populus euphratica* along the downstream river banks [11]. The response scope of the vegetation was gradually enlarged due to the rise of the groundwater level. In fact, only some flooding reached the Taitema Lake. However, it helped increase the water surface area to 415.67 km^2^ by 2014 and prevented it from desiccation [12]. It was regarded as one of the most remarkable examples of recovery achievements in arid regions. Therefore, the Heihe River and Shiyang River have adopted similar approaches to restore the ecosystems of lower reaches and terminal lakes and numerous scholars analyze the ecological effects of the water transport from different angles. In general, many studies have presented that ecological water transport plays a promoting role in raising the groundwater level of the lower reaches and the terminal lakes, increasing the vegetation richness and wetland area, and prolonging the growing season of riparian vegetation [13,14,15].

However, in previous studies, when assessing the influence of water transport on terminal lakes, especially the response of groundwater level to water delivery, scholars usually used the data from one observation well or set up certain observation points in a part of the region to approximately illustrate the spatial distribution of the groundwater level of the entire region. Thus, there may be a lack of investigation into the overall spatial distribution patterns of groundwater levels in terminal lakes after water transportation.

Hence, in order to analyze and evaluate the ecological effects of water transport to lakes in arid regions more comprehensively, Qingtu Lake was selected as the study area in this paper to analyze the water surface area, spatial distribution of groundwater, vegetation coverage, and the quantity as well as spatial distribution of typical vegetation before and after water transport. The specific analysis process was mainly presented in the following three aspects. Firstly, the remote sensing images from 2010 to 2018 were used to compute the Normalized Vegetation Difference Index (NDVI) of whole vegetation around Qingtu Lake in order to determine the temporal changes in vegetation coverage since water transport began. Meanwhile, the spatial distribution changes in vegetation coverage around Qingtu Lake before and after water supply were analyzed through a comparison of the spatial distribution of regional vegetation coverage in 2010 and 2018. Secondly, by using the supervised classification, the water surface area of Qingtu Lake in 2018 after water transportation and the changes in the quantity and distribution of *Phragmites australis* and *Nitraria tangutorum* communities in 2010 and 2018 were obtained. Finally, the spatial distribution of groundwater depth in Qingtu Lake after water supply was analyzed by spatially interpolating the field investigation data of groundwater depth. This study complements the deficiency of systematic analysis of spatial distribution patterns of groundwater levels after water delivery in the past when evaluating the ecological effects of ecological water transportation. Moreover, the results provide a scientific basis and data support for assessing the ecological effects of water transport to Qingtu Lake, optimizing the water transportation scheduling scheme and improving the restoration efficiency of the Qingtu Lake ecosystem.

## 2. Study Area

**Figure 1:**
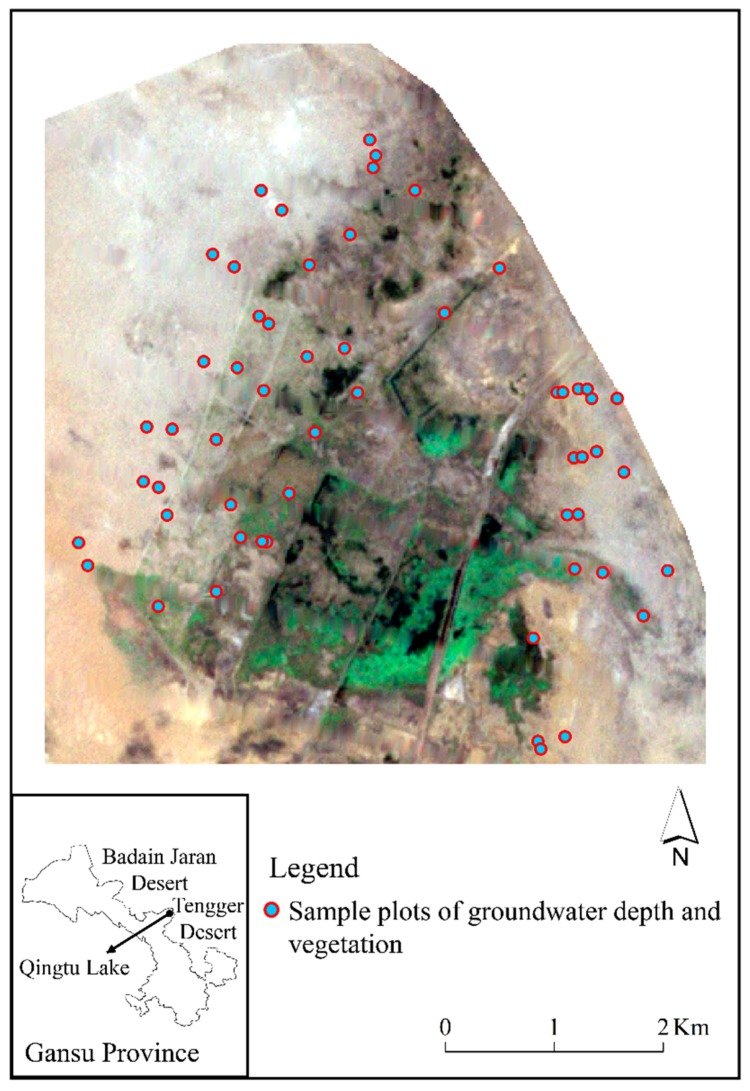
Study area and sample plots.

Qingtu Lake, the terminal lake of the Shiyang River, is located 70 km northeast of Minqin County, Gansu Province, China. With geographical coordinates of 39°04′–39°10′ N and 103°35′–103°38′ E, it is adjacent to Tengger Desert in the east and Badain Jaran Desert in the northwest (Figure 1). The region is located at the edge of the East Asian monsoon with a temperate continental arid desert climate. This region has an average of 110 mm of precipitation and 2640 mm of evaporation annually, which is 24 times the former. In total, 73% of the annual precipitation occurs between July and September, and the annual average temperature of the region is 7.8 °C [16]. The annual sunshine duration is 3181 h, and the frost-free period in this region is 168 d. Gray-brown desert soil is the zonal soil and intra-zonal soil is meadow-boggy soil and aeolian sandy soil [17]. The main regional vegetation is *P. australis*, *N. tangutorum*, *Suaeda glauca*, *Haloxylon ammodendron*, *Kalidium foliatum*, etc.

Owing to its unique geographical location, Qingtu Lake is an important ecological barrier between the two deserts. In the early 20th century, Qingtu Lake covered an area of 120 km^2^ [18]. However, since the 1950s, due to the increasing amount of water consumption in the upper and middle reaches of the Shiyang River, especially the construction of the Hongyashan reservoir, the inflow volume of the lake has continually decreased. As a result, the lake gradually shrank and dried up in 1959. Consequently, most of the region was covered by shifting sand, which accelerated the converging of the two deserts. Hence, in order to restore the regional ecological environment and enhance the ecological function of Qingtu Lake, the government decided to transfer ecological water from the Hongyashan reservoir to Qingtu Lake through the artificial channels annually since September 2010.

## 3. Data and Methodologies

**Figure 2:**
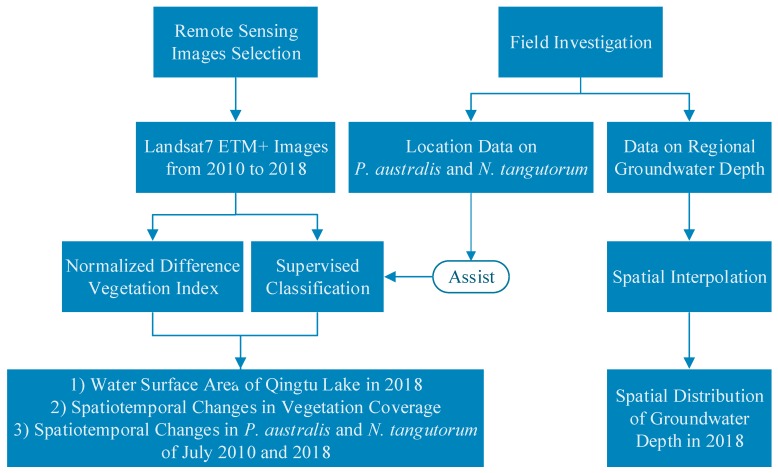
Study technology roadmap.

Figure 2 shows the thinking behind the research premises of this study. In general, the study was mainly divided into two parts: remote sensing interpretation and field investigation. Firstly, by selecting the remote sensing images before and after the water transport, NDVI was calculated, and supervised classification was carried out to obtain the water surface area in 2018, the spatial and temporal changes in vegetation coverage, as well as spatiotemporal variations in the *P. australis* and *N. tangutorum* communities of Qingtu Lake in 2010 and 2018. Secondly, the data on the regional groundwater depth and the location data on the *P. australis* and *N. tangutorum* communities in 2018 were obtained by field investigation. The former was then used to obtain the spatial distribution of groundwater depth through spatial interpolation and the latter was applied to assist in the completion of supervised classification to improve the accuracy of the results. The details of the methods are described as follows.

### 3.1. Field Investigation

The field investigation proceeded from July to August in 2018. A total of 56 sample plots, located from north to south and from the waterside to desert edge on both sides of the lake, were investigated in the spatial distribution of the regional typical hygrophyte *P. australis* and typical xerophyte *N. tangutorum* as well as the groundwater depth (Figure 1).

We first undertook the vegetation survey. All sample plots were determined by the vegetation survey which was based on presence–absence sampling [19]. The size of each plot was 20 × 20 m. During the survey, registrations were made only in terms of whether or not the *P. australis* or *N. tangutorum* communities were present in a plot when investigated from waterside to desert edge. Registrations were mainly divided into four cases: (1) presence of *P. australis* and absence of *N. tangutorum* in a plot; (2) presence of *P. australis* and *N. tangutorum* in a plot; (3) absence of *P. australis* and presence of *N. tangutorum* in a plot; and (4) absence of *P. australis* and *N. tangutorum* in a plot. Moreover, each case was recorded only once and was not repeated for each time of survey. The information on the type of vegetation and its location was used to assist subsequent remote sensing interpretation to obtain the spatial distribution patterns of *P. australis* and *N. tangutorum*.

Furthermore, the groundwater depth data were obtained by excavating to the groundwater table in the center of each sample plot. Therefore, by using the Kriging interpolation module in the Arcgis software, the Ordinary Kriging method [20] was selected to interpolate the data to obtain the spatial distribution of groundwater depth. In addition, the areas without water surface cover were extracted for a mask which was applied to the spatial interpolation results. Therefore, the spatial distribution of groundwater in Qingtu Lake only considered the region without water surface cover.

### 3.2. Remote Sensing Interpretation

#### 3.2.1. Remote Sensing Images Selection and Pre-Processing

Remote sensing images of July or August in each year between 2010 and 2018 were used for a comparative analysis. Every image was selected from the U.S. Landsat 7 satellite. With Enhanced Thematic Mapper Plus sensors, it was able to provide panchromatic and multispectral images at 15 m and 30 m resolutions, respectively. There were mainly two considerations of the images’ selection. It aimed to eliminate the influence of errors caused by the growth differences of vegetation in different growth periods when comparing the changes of vegetation before and after the water transportation by supervised classification. Additionally, it aimed to match the investigation period during which the survey data could be used to assist the classification in order to improve its accuracy.

The images were firstly pre-processed with ENVI 5.3 (Harris Geospatial Solutions, Inc., Broomfield, CO, USA), which included radiometric calibration, layer stacking, atmospheric correction, destriped Thematic Mapper data, image fusion and cropping. The Landsat calibration module in the ENVI software was used to read the associated parameters for radiometric calibration through referring to the head file. Moreover, the Fast Line-of-sight Atmospheric Analysis of Spectral Hypercubes (FLAASH) atmospheric correction module was applied for the atmospheric correction of images that were based on the Moderate Resolution Atmospheric Transmission model. In addition, false color composite images of Band 4, Band 3 and Band 2 of Enhanced Thematic Mapper Plus (ETM+) images of the study area were then obtained with a resolution of 15 m by fusing the images.

#### 3.2.2. Normalized Difference Vegetation Index and Supervised Classification

NDVI is widely applied to detect the vegetation growth state, reflect the coverage and yield of vegetation, as well as monitor biomass [21,22,23,24]. Since the NDVI data of every space point have high comparability and temporal consistency, the spatial and temporal change in terrestrial biomass can be effectively reflected by analyzing the spatiotemporal variation in NDVI. Therefore, in this study, the NDVI of the study area was calculated using the NDVI module in ENVI 5.3 to obtain the change in regional vegetation coverage from 2010 to 2018 and the variation in the spatial distribution of vegetation in 2010 and 2018.

Furthermore, based on the location information of water surface and typical vegetation that was obtained from the field survey, the supervised classification was used to classify the water surface, *P. australis* and *N. tangutorum* communities to obtain their spatial distribution. Supervised classification is a commonly used statistical decision classification method with high accuracy. Various training samples are extracted from the training ground with known classes. By selecting characteristic variables, determining discriminant functions or discriminant rules, every pixel in the image is classified into each given class [25].

The specific method is as follows: firstly, based on the false color composite images of the study area in 2010 and 2018, combined with the location information of different land cover obtained from the field survey, the feature judgment of images was conducted through visual inspection. Four types of land covers were distinguished: water surface, *P. australis*, *N. tangutorum* and others. Secondly, we used the Region of Interest (ROI) tool in ENVI to select training samples for each class on the images. In this step, typical vegetation, water surface and other locations collected in the field investigation were input into the ROI tool as part of the training samples. By referring to these locations, training samples of various land covers were supplemented by visual inspection. The combination with field investigation may overcome some restrictions, such as the limitation of the image resolution, which sometimes makes it difficult to distinguish between different vegetation in some areas more clearly and accurately, so as to improve the accuracy of the classification as much as possible. Thirdly, the Maximum Likelihood Classification classifier was used for classification. It assumes that the statistic of each class in each band is normally distributed and calculates the likelihood that a given pixel belongs to a training sample. The pixel is eventually merged into the class with the maximum likelihood [26]. Subsequently, class statistics were performed on the classification results, and statistical information such as the area of the water surface, *P. australis*, and *N. tangutorum* were obtained and recorded. Finally, the accuracy of the classification was evaluated by establishing a confusion matrix. The overall accuracy of classification in 2010 and 2018 was 98.98% and 98.37%, respectively. In addition, both Kappa coefficients reached 0.97. This indicated that the results were adequate, which ensured the accuracy of the data. Overall, through the above methods, the temporal and spatial variations in water and vegetation were analyzed.

## 4. Results and Discussion

### 4.1. Water Surface Area and Spatial Distribution of Groundwater Depth after Water Transport

#### 4.1.1. The Water Surface Area of Qingtu Lake in 2018

**Figure 3:**
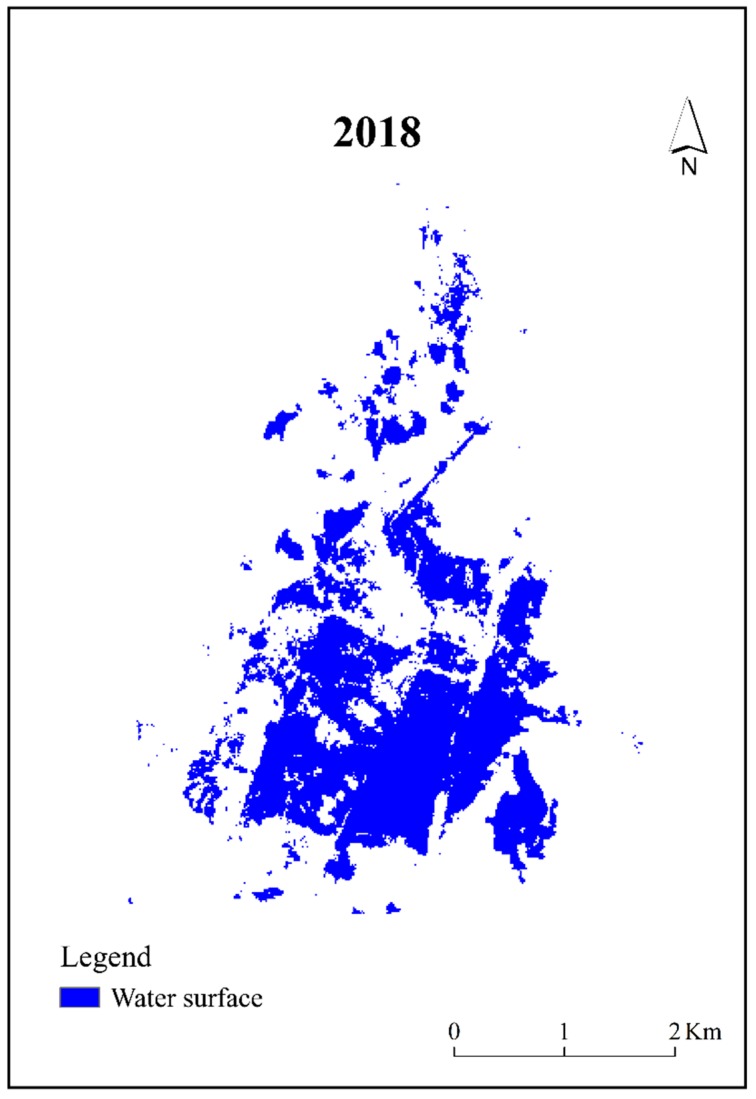
Water surface of Qingtu Lake in July 2018.

Figure 3 shows the water surface of the lake in July 2018. Since the lake dried up in 1959 and the water transport started in September 2010, the surface area in July 2010 was zero. By July 2018, the water surface area increased to about 5.68 km^2^. Since the water was transported into the area from the south on both sides of the east and west through artificial channels, the water surface formed in the south was more continuous, and the total area was larger than that in the north.

Chen et al. [18] state that due to the continuous water transport, the surface area of Qingtu Lake increased year by year, reaching 10.82 km^2^ in 2016. However, as of July 2018, the water surface area was 5.68 km^2^ according to the results in this study. In the study of the ecological effects evaluation for short-term planning of the Tarim River, Zhang et al. [12] found that when the water supply reached the terminal lake in successive years, the water surface of Taitema Lake was gradually formed and expanded. Nevertheless, the surface area shrank in 2005 because the water volume did not meet the requirements. Furthermore, due to the ecological effects, water did not reach the Taitema Lake from 2006 to 2009. It had completely dried up. After the adjustment in 2010, the water surface recovered again. This indicated that water transportation plays an important role in promoting the formation of the water surface of terminal lakes in arid regions, while the surface area was unstable. The interruption of the water supply will cause the water surface to shrink rapidly or even dry up. Therefore, the decrease in the water surface area of Qingtu Lake in July 2018 may be related to the water transport mode in that the inter-annual implementation is continuous, whereas the intra-annual implementation is concentrated and intermittent. Moreover, under the premise of reaching the total water requirements, this type of water transport may cause the water surface area to fluctuate within a year but will not cause it to dry up. Hence, it was necessary to transfer a certain amount of water into the lake each year in order to maintain the water surface that has been restored.

#### 4.1.2. Spatial Distribution of Groundwater Depth in 2018

**Figure 4:**
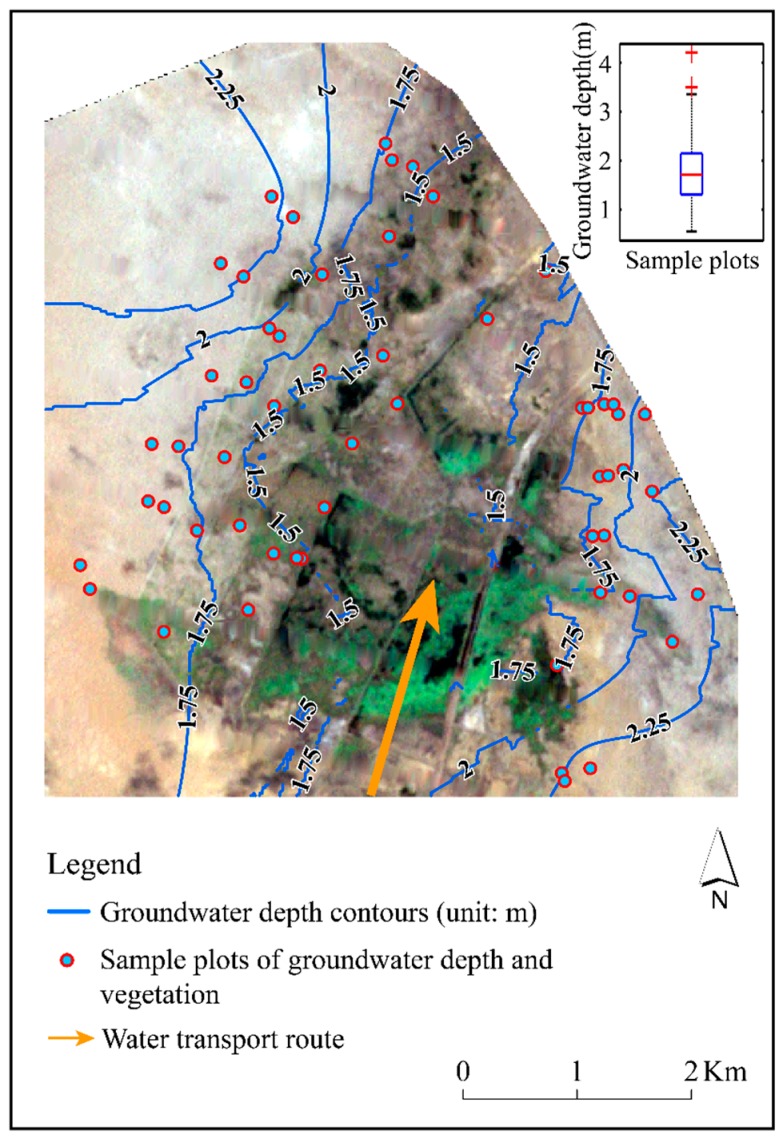
Spatial distribution of the groundwater depth of Qingtu Lake in July 2018.

There was a spatial gradient variation in regional groundwater depth that it increased in gradient from the waterside to the desert edge, which is shown in Figure 4. Compared with the average groundwater depth of 3.91 m of Qingtu Lake in 2008 [18], the water transportation had a significant effect on reducing the groundwater depth. Besides, the formation of this kind of pattern may be closely related to the form of water transportation which was planar. The groundwater of regions receiving water directly from the lake was mainly supplied by vertical infiltration—the recharge was direct and sufficient, and the groundwater depth reduction was the largest. With the continuous infiltration in these regions, the lateral infiltration occurred at the same time. This would decrease with an increase of horizontal distance. The spatial difference in the groundwater lateral infiltration supply would result in the formation of such spatial patterns over time.

### 4.2. Changes in Vegetation around Qingtu Lake

#### 4.2.1. Spatiotemporal Changes in Vegetation Coverage

**Figure 5:**
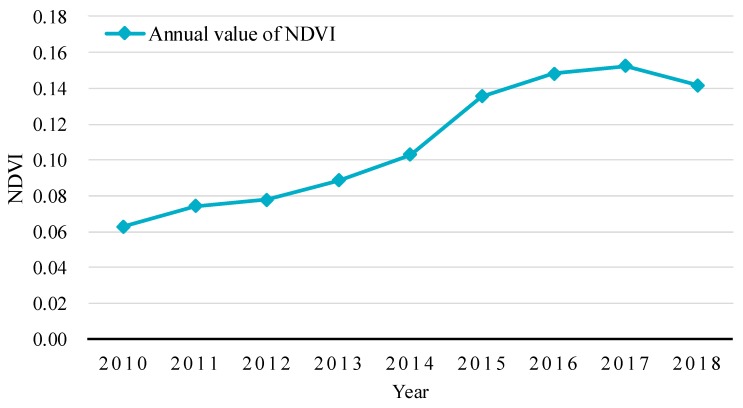
Change in vegetation coverage around Qingtu Lake from 2010 to 2018. NDVI: Normalized Vegetation Difference Index.

Through the calculation of the annual NDVI of the study area from 2010 to 2018, the overall regional vegetation coverage had an obvious increasing trend since water transport began (Figure 5). In 2010, before the water delivery, the mean value of NDVI was about 0.06. In the next five years, the regional vegetation coverage increased rapidly, especially from 2014 to 2015, when the NDVI increased from approximately 0.10 to 0.14. Since 2015, the growth of NDVI has slowed down to reach a maximum in 2017, which was about 0.15. In 2018, it decreased slightly to nearly 0.14. However, compared with 2010, the growth of regional vegetation coverage was still significant.

**Figure 6:**
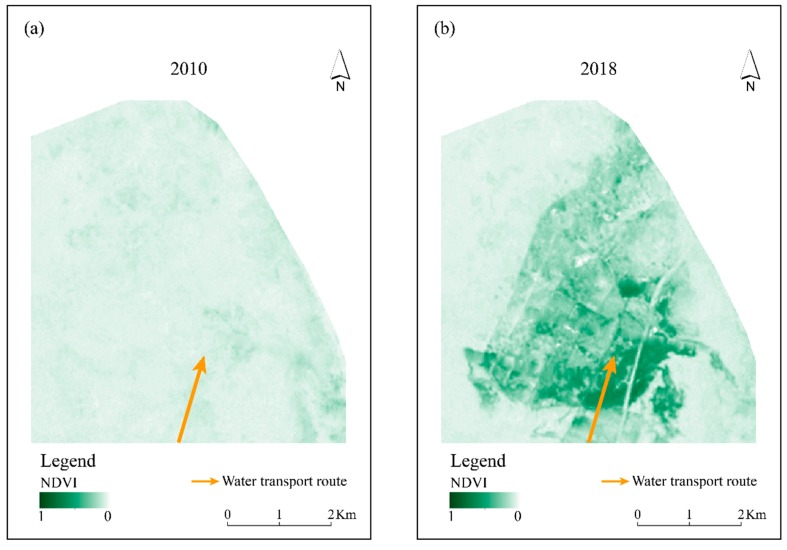
Panel (**a**): variation in the Normalized Vegetation Difference Index (NDVI) (scale from 0 to 1) of Qingtu Lake in July 2010. Panel (**b**): variation in the NDVI (scale from 0 to 1) of Qingtu Lake in July 2018.

Furthermore, Figure 6a,b illustrate the spatial variation in the overall vegetation coverage around Qingtu Lake before and after water transportation. Vegetation coverage in the south was generally superior to that in the north in 2018, which may be relevant to the location to which the water was transported. Besides, combined with the results of water surface classification, it was found that large areas of vegetation were concentrated in shallow waters and adjacent regions. Thus, it can be inferred that the ecological water transportation promoted the overall vegetation growth of the Qingtu Lake desert oasis, particularly the vegetation in shallow waters. The formation of the water surface promoted more hygrophytes to grow in the shallow water area and the lake’s surrounding areas with high moisture, thus replacing the original xerophytes.

#### 4.2.2. Spatiotemporal Changes in *P. australis* and *N. tangutorum* Communities around Qingtu Lake

Through statistical calculation of the supervised classification results, the areas of *N. tangutorum* and *P. australis* were 13.47 km^2^ and 0.19 km^2^ in July 2010, respectively. *N. tangutorum* was the dominant species around Qingtu Lake. By July 2018, compared with 2010, the area of *N. tangutorum* increased slightly to 13.95 km^2^, while the area of *P. australis* increased significantly by nearly 54 times to reach 10.18 km^2^. This indicated that the water transportation had an overall promoting effect on the growth of vegetation in the study area but showed an obvious difference in the promotion degree of different growth types. This may be associated with the decline in some xerophytes due to their submergence in water that inhibited the root respiration.

**Figure 7:**
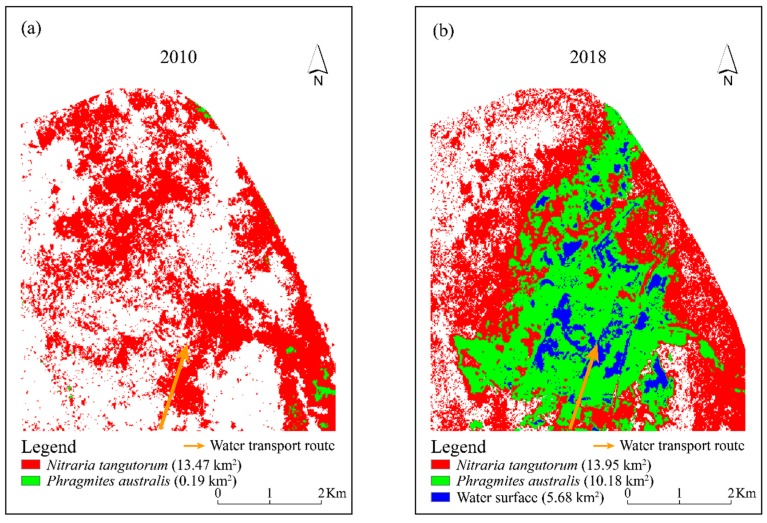
Panel (**a**): variation in *N. tangutorum* and *P. australis* distribution of Qingtu Lake in July 2010. Panel (**b**): variation in *N. tangutorum*, *P. australis* and water surface distribution of Qingtu Lake in July 2018.

**Figure 8:**
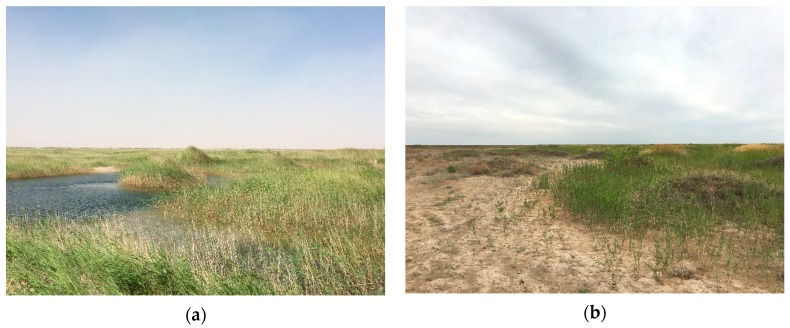
Panel (**a**): *P. australis* distributed in the shallow waters of Qingtu Lake; Panel (**b**): vegetation transitions from *P. australis* to *N. tangutorum* (Photograph: X.C., July 2018).

Figure 7a,b illustrate the spatial distribution of *P. australis* and *N. tangutorum* before and after the water transportation. Before the water transportation began, the *N. tangutorum* communities showed a large continuous distribution, while only a few *P. australis* communities were distributed continuously in the north and southeast which may be related to the local topography and soil structural composition, and the rest were distributed sporadically in the study area. In 2018, *N. tangutorum* communities that originally grew in the existing waters disappeared. Instead, numerous *P. australis* communities grew within and near waters (Figure 8a), with a small amount of *N. tangutorum* symbiosis with it. Moreover, from the water to the desert edge, the spatial distribution density of *N. tangutorum* showed a trend that went from dense to sparse.

The results indicated that the distribution pattern of the vegetation has changed from a continuous distribution of a large area of xerophytes to a gradient distribution that transitioned from hygrophytes to xerophytes. The change in the vegetation spatial pattern was mainly related to the formation and distribution of water surface. Qingtu Lake was in an arid desert environment before water transport. Meanwhile, *N. tangutorum* has the characteristics of extreme drought tolerance, which is an important species of desert and semi-desert grassland [27]. Thus, this natural environment was conducive to the growth of *N. tangutorum*, making the *N. tangutorum* become the main building species in this area [28]. However, with the gradual formation of water surface, some of the *N. tangutorum* sand dunes were submerged by water, which inhibited the respiration of the roots of the *N. tangutorum*, or the leaching salt moved down and damaged the root system, resulting in a decline in numbers and death [18]. As a result, the original *N. tangutorum* in the water surface formation area was replaced by *P. australis*. Meanwhile, as the water transport proceeded, the groundwater depth was gradually reduced and formed a spatial distribution pattern with an increasing gradient. Chen et al. [1] pointed out in their research that the leaf characteristics of *P. australis* were sensitive to the fluctuations of groundwater depth. This indicated that the growth of *P. australis* communities was not only influenced by the formation of the water surface, but also closely associated with the change in groundwater depth. Hence, a large number of *P. australis* have also grown in areas with shallow groundwater depth around the waters. With the increasing of groundwater depth, the dominant species of vegetation changed from *P. australis* to *N. tangutorum* (Figure 8b). Furthermore, the roots of *N. tangutorum* were mainly horizontal roots and oblique roots, and its effective roots were mostly concentrated in shallow soil [29]. During the field investigation, it was found that the horizontal roots of *N. tangutorum* were extremely developed, the length of which was generally 2–3 m, and most of them were distributed in the shallow soil 10–70 cm below the surface. Therefore, in the areas close to the lake and with relatively shallow groundwater depth, *N. tangutorum* mainly absorbed water through its developed horizontal roots and grew in large quantities. With the increase of groundwater depth, the growth of *N. tangutorum* that distributed far from the waterside was mainly dependent on the vertical root absorption of groundwater. The groundwater that can be utilized by *N. tangutorum* may decrease, resulting in the decrease of population density. Thus, it can also be inferred that there was a certain spatial synchronization between the spatial distribution of vegetation and the groundwater depth of Qingtu Lake.

**Figure 9:**
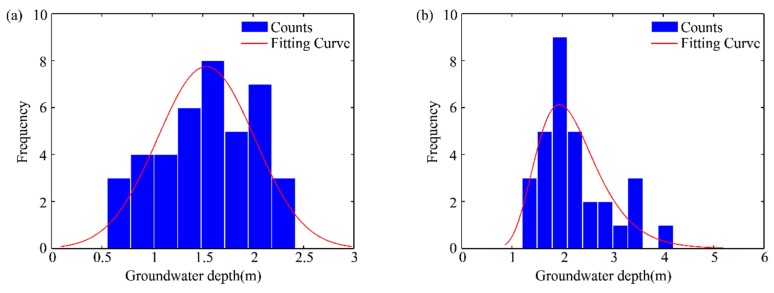
Panel (**a**): the relationship between groundwater depth and the growth frequency of *P. australis*; Panel (**b**): the relationship between groundwater depth and the growth frequency of *N. tangutorum*.

Through the attempt to analyze the correlation between the growth of the two typical vegetation types and the groundwater depth of Qingtu Lake, it was found that *P. australis* data and *N. tangutorum* data conformed to the normal distribution and the log-normal distribution, respectively. The distribution curves were fitted to the data, which are shown in Figure 9a,b and Table 1. It showed that when the groundwater depth was about 1.53 m and 1.94 m, the frequency of *P. australis* and *N. tangutorum* reached the maximum, respectively. This indicated that the groundwater depth of 1.53 m and 1.94 m may be more suitable for the growth of *P. australis* and *N. tangutorum*. Furthermore, it may be further demonstrated that the regional distribution pattern of typical vegetation was closely related to the spatial variation in the groundwater depth which was mainly affected by the water transport.

## 5. Conclusions

In this study, based on the field investigation and the remote sensing interpretation, the ecological effects of water transport to Qingtu Lake were assessed. The results are as follows:

1. Initially, when the water transport was set up in September 2010, Qingtu Lake gradually reformed a large continuous surface, with an area of 5.68 km^2^ by July 2018. The water transport replenished the local groundwater and the lake formed a spatial gradient distribution in groundwater depth. The depth increased in gradient from the waterside to the desert edge.
2. The overall vegetation coverage around Qingtu Lake increased significantly since water transport began, especially from 2014 to 2015, while it decreased slightly in 2018, with the NDVI reaching 0.14. In addition, after water delivery, the vegetation in the study area was more distributed in shallow waters and its surrounding areas.
3. There was a slight increase in *N. tangutorum* communities in 2018 compared with numbers in 2010. However, the area where *P. australis* communities are found increased by approximately 10 km^2^. In addition, from the waterside to the desert edge, the vegetation showed a gradient distribution in which the species transitioned from hygrophytes to xerophytes. It can be concluded that the water transport was beneficial to the restoration of the regional vegetation and the ecological environment, with good ecological effects.

The results of this study can provide data support for subsequent studies on the determination of ecological water requirements of Qingtu Lake—this will reveal how much ecological water is needed to maintain a certain water surface area and vegetation growth area. Meanwhile, it also provides scientific evidence for a comprehensive assessment of the ecological effects of water transport to Qingtu Lake. Meanwhile, it has important reference significance for the protection and restoration of other lakes in arid regions.

However, in this study, there are still some limitations. The first is that there may be too few sample plots for the statistical analysis to accurately obtain the most suitable groundwater depth intervals for the two kinds of plants. Second, due to the limitation of the remote sensing images resolution, there may be some deviations in the interpretation of vegetation in some local areas. Third, the results of the spatial interpolation were affected by such factors as the number of sample plots, their spatial distribution, and the interpolation methods. Due to the number and the selection of the spatial distribution of sample plots, they may be insufficient or uneven. The interpolation results of groundwater depth in some local areas may have some deviations. In addition, the factors affecting the distribution of vegetation were not considered comprehensively. This paper only considered the single factor of water. However, it did not consider other possibly related factors such as soil texture and salinity. Moreover, because of the limitation of the existing observation conditions, it was difficult to make continuous observations of vegetation and groundwater in the study area, which may mean that the statistical analysis of the results is too weak to support the conclusions regarding spatiotemporal variations. Hence, we may consider addressing these issues in future research and try to make long-term continuous observations of vegetation and groundwater variations in the study area. In the next stage, the study will focus on exploring the correlation between water transport volume and water surface area, the relationship between typical vegetation and the distribution of water and salt in the soil, and whether the soil texture also affects the formation of regional vegetation distribution.

## Figures and Tables

**Table 1:** Parameters of normal distribution and log-normal distribution fitting curves of *P. australis* and *N. tangutorum*.

Typical Vegetation	*μ*/m	*σ*/m	Fitting Curves Equations
*P. australis*	1.53	0.48	$f(x)=\frac{1}{0.48\sqrt{2\pi }}e^{\frac{-{\left(x-1.53\right)}^{2}}{0.46}}$
*N. tangutorum*	0.75	0.30	$f(x)=\frac{1}{0.3x\sqrt{2\pi }}e^{\frac{-{\left(\ln x-0.75\right)}^{2}}{0.18}}$
